# Segmenting with confidence through uncertainty quantification for brain tumor imaging

**DOI:** 10.1038/s41746-026-02902-0

**Published:** 2026-06-19

**Authors:** Yassine Guennoun, Pierre Nedelec, Mark McArthur, Evan Bloch, Jinchi Wei, Leo Sugrue, Evan Calabrese, Andreas M. Rauschecker

**Affiliations:** 1 Center for Intelligent Imaging (ci2), Department of Radiology & Biomedical Imaging, University of California San Francisco (UCSF), San Francisco, CA USA; 2 Department of Radiology, Duke University Medical Center, Durham, NC USA

**Keywords:** Computational biology and bioinformatics, Health care, Medical research, Neurology, Neuroscience, Oncology

## Abstract

A major barrier to clinical adoption of artificial intelligence (AI) for brain tumor monitoring is the lack of calibrated uncertainty in automated segmentation, limiting clinician trust. We developed a deep learning framework that generates uncertainty estimates for meningioma segmentation on brain MRI. Evidential deep learning ensembles were trained on 1655 post-contrast T1-weighted MRIs (788 patients) to capture aleatoric-like and epistemic-like uncertainty. Architecturally homogeneous and heterogeneous ensembles were evaluated on an independent test set of 68 MRIs (43 patients) and compared with existing methods. Performance was assessed using Dice similarity coefficient, spatial agreement between uncertainty maps and neuroradiologist-identified ambiguous regions, and calibration of volumetric credible intervals. The model achieved high accuracy (median Dice 0.93), with uncertainty maps aligning with ambiguous regions and well-calibrated volume estimates. External validation in 353 patients confirmed generalizability (median Dice 0.92), supporting safer clinical AI deployment and enabling calibrated uncertainty estimation for lesion segmentation beyond meningiomas.

## Introduction

Medical imaging is central to the practice of medicine, allowing experts to localize, diagnose, and longitudinally follow abnormalities inside the body. For example, in neuro-oncology practice, MRI-based monitoring of brain tumors, such as meningiomas, guides critical treatment decisions. The clinical standard for measuring tumors often relies on either subjective evaluation or simplified bi-dimensional measurements as outlined by the Response Assessment in Neuro-Oncology (RANO) working group, primarily designed for gliomas (Wen et al.^1^) and extended to meningiomas^1,2^. However, for tumors with slow or irregular growth patterns, these 2D metrics can fail to capture the true tumor burden, with studies showing that 3D volumetric analysis provides a more accurate picture of progression^3,4^.

While deep learning has demonstrated strong performance metrics for 3D segmentation across a wide variety of biomedical image segmentation tasks, clinical adoption of these tools has been limited. A primary reason for limited adoption is a lack of trust and interpretability in overconfident deterministic models. Standard segmentation models produce deterministic outputs that mask the inherent uncertainty of the prediction, making it difficult for clinicians to distinguish true biological growth from measurement noise. Although similar implicit uncertainty is also present in human-generated segmentations due to inter-observer variability^5^, such uncertainty is rarely quantified or calibrated at the voxel level in routine clinical practice. Furthermore, small segmentation inaccuracies near eloquent brain regions or along tumor margins could have substantial consequences for radiation or surgical planning, or for evaluation of tumor growth versus stability, directly affecting treatment decisions.

Until mechanisms exist to quantify and communicate voxelwise model confidence in individual patients, segmentation methods will remain limited in terms of clinical applicability. Model precision, and its confidence, can vary by tumor pathology, across tumors of the same pathology in different locations or with different imaging appearances, and within portions of a single tumor. Uncertainty quantification (UQ)^6^ is a group of methods for addressing this inherent variability in model confidence. Techniques include: Bayesian methods such as Monte Carlo Dropout^7^ and Deep Ensembles^8^ for capturing model uncertainty, and generative approaches like the Probabilistic U-Net^9^ for modeling structural ambiguity in segmentations. A more recent paradigm is Evidential Deep Learning (EDL), which directly models evidence from data to form a predictive distribution^10,11^. However, recent work has shown that single-model EDL can produce unreliable uncertainty estimates, motivating the need for more robust, ensemble-based approaches^12^.

The purpose of this study was to develop and validate a fully automated framework for tumor segmentation on real clinical brain MRIs, built on ensembles of EDL models, capable of producing clinically meaningful volumetric credible intervals and interpretable uncertainty maps. We focus this study on meningiomas, the most common primary brain tumor, accounting for over one-third of all intracranial tumors and nearly half of all primary brain tumors^13–15^. While some meningiomas have very well-defined borders and model confidence would be expected to be high, others are infiltrative, adjacent to anatomical structures that obscure the tumor boundary, or demonstrate unusual shapes, all leading to expected regions of model uncertainty that are critical to consider when measuring tumor volumes or when communicating tumor boundaries to clinicians. Notably, our study also includes post-operative brain MRIs; uncertainty can be especially high when treatment-related changes exhibit enhancement patterns similar to tumor tissue, yet these MRIs are critical for evaluating growth of residual or recurrent tumors^16^. We hypothesized that the clinical reliability and calibration of these uncertainty estimates are critically dependent on architectural heterogeneity and that they directly relate to radiologists’ uncertainty of tumor boundaries.

## Results

**Fig. 1:**
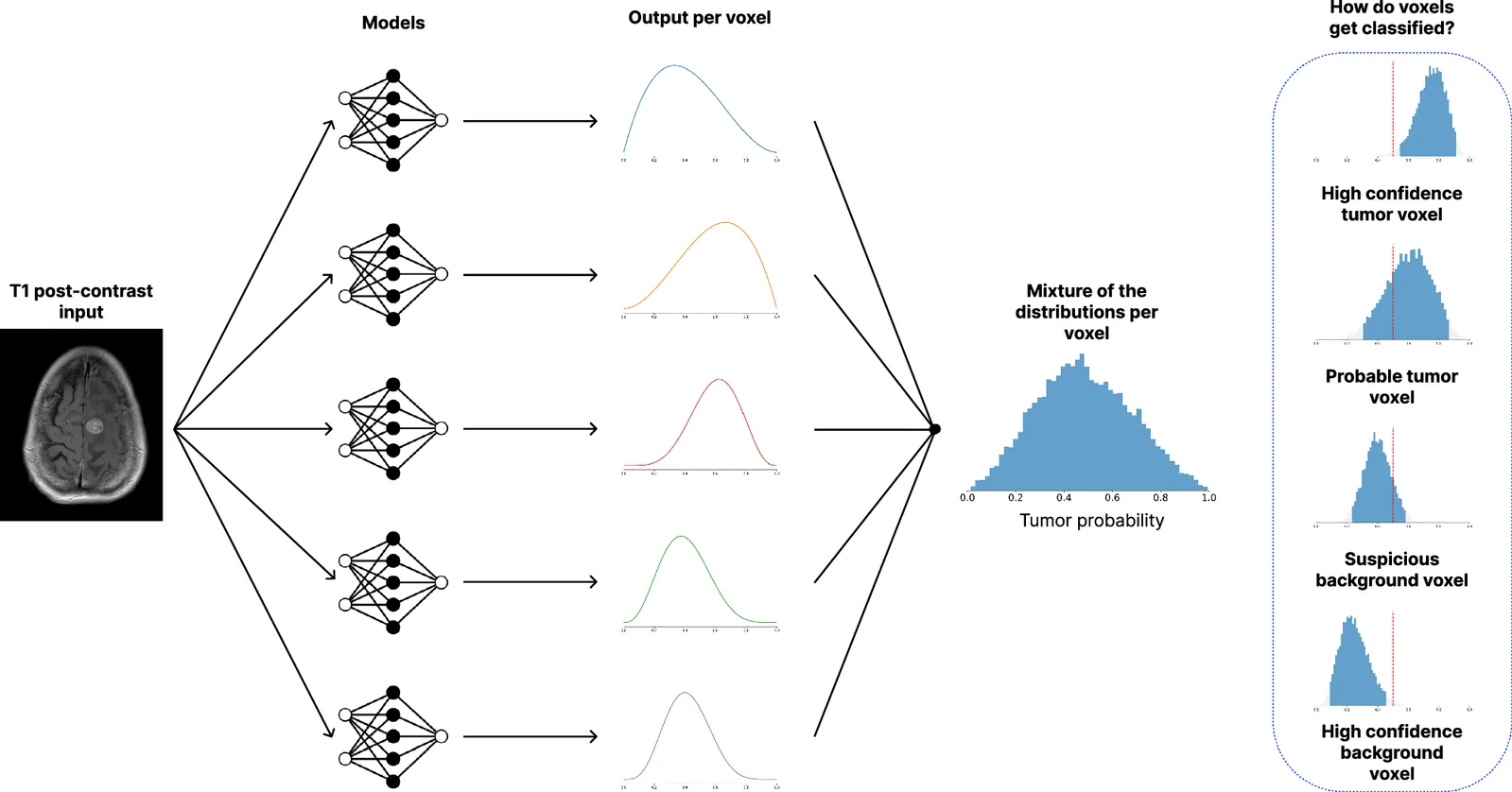
Conceptual overview of the evidential deep learning ensemble framework. A T1 post-contrast MRI is processed by an ensemble of M=5 models. Each model outputs the parameters of a Beta distribution per voxel, representing the probability of tumor presence. These individual distributions are combined into a mixture model, from which a final posterior probability is derived to classify each voxel and quantify uncertainty.

An evidential deep learning (EDL) ensemble framework was trained to detect and segment meningiomas based on T1-weighted post-contrast images acquired as part of routine clinical care (Fig. 1). Importantly, as described in detail in Methods, the EDL framework allows for estimation of the probability of tumor presence at each voxel, translating into a measure of voxel-wise uncertainty and overall uncertainty of tumor volume. Further details of model training can be found in the Methods section.

### The EDL framework models demonstrate state-of-the-art segmentation performance

**Fig. 2:**
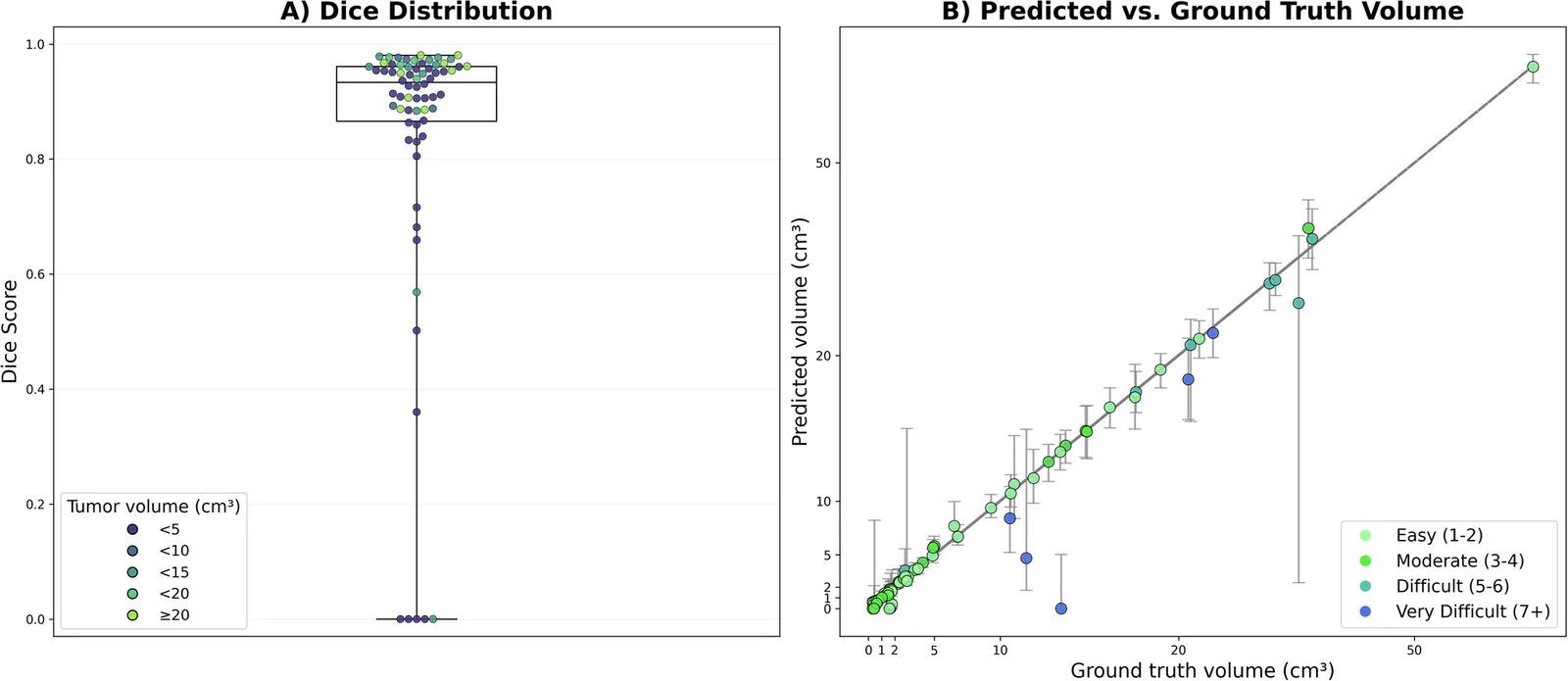
Quantitative evaluation of the heterogeneous EDL ensemble on the test set (*N* = 68). **A** Distribution of Dice scores, stratified by ground truth tumor volume. The model maintains high performance across a range of tumor sizes. The few zero-Dice points correspond to challenging or out-of-distribution cases: the green dot reflects the most difficult example (rated 9/10), while the others are small 4 cm³ tumors. **B** Predicted versus ground truth volume, colored by radiologist-assessed difficulty. Error bars indicate the 95% credible interval derived through the EDL framework. The credible interval widens for smaller and more difficult tumors, demonstrating appropriate uncertainty calibration.

For the test set, we established a strict human reference standard where every case was manually segmented by a neuroradiologist. We then compared the segmentation performance of three ensemble configurations against this ground truth. A baseline homogeneous ensemble (five SegResNets without EDL) achieved a median Dice of 0.93. Our proposed homogeneous EDL ensemble (five SegResNets) scored a median Dice of 0.95, while our heterogeneous EDL ensemble (mixed-architecture) scored a median Dice of 0.93 (Fig. 2). The corresponding distribution plots for the other ensemble configurations are provided in Figs. S1–S3 in the Supplementary Materials, presented in the same format as Fig. 2 for direct comparison.

A repeated-measures ANOVA revealed a significant effect of the ensemble type on Dice scores (p<0.001), with the homogeneous ensemble outperforming both heterogeneous and baseline configurations. Both models outperformed a baseline 5-fold ensemble nnU-net v2 model^17^ trained on the same dataset, which achieved a median Dice score of 0.77 (*p* < 0.05). These results demonstrate that the EDL framework, in both its homogeneous and heterogeneous implementations, provides uncertainty quantification without compromising segmentation accuracy.

While all five individual architectures in the mixed ensemble achieve a similarly high median Dice score, they possess different and often uncorrelated failure modes. We observed instances where the SWIN UNETR and DiNTS failed while the SegResNets succeeded, and vice versa.

### External validation demonstrates high generalizability of the model

To assess generalizability beyond our private internal dataset, we evaluated all four configurations on an independent external cohort of 353 patients with meningiomas obtained from Duke University. The homogeneous EDL ensemble achieved a median Dice score of 0.92, not significantly different than the performance observed on the internal (UCSF) test set (*p* = 0.57, independent *t*-test), demonstrating cross-institutional robustness and consistency. Similarly, the Mixed EDL configuration achieved a median Dice of 0.93 (*p* = 0.71), while MC Dropout (*p* = 0.2 and *p* = 0.5) both achieved median Dice scores of 0.92 (*p* = 0.63 and *p* = 0.56, respectively). A detailed analysis of the homogeneous EDL ensemble’s performance as a function of tumor volume is provided in the Supplementary Material, where Dice scores are plotted against ground truth volume to visualize performance stability across tumor sizes (Figs. S4 and S5).

### EDL framework improves uncertainty quantification over prior methods

Beyond segmentation accuracy, we analyzed the quality of the predictions and uncertainty estimates. Empirical coverage refers to the percentage of cases where the true ground-truth volume falls within the model’s predicted 95% credible interval. Thus, a 95% credible interval should ideally result in empirical coverage of 95%.

The results (Table 1) reveal differences in uncertainty quality across methods, which we analyze by first comparing our two proposed EDL ensembles and then benchmarking against MC Dropout^7^.

**Table 1:** Empirical coverage and Pearson correlations between predicted 95% credible interval width and segmentation metrics (segmentation error, average symmetric surface distance, and volumetric error) across four different frameworks

Method	Coverage	Seg. error (${R}^{2}$)	ASSD (${R}^{2}$)	Vol. error (${R}^{2}$)
Homogeneous EDL (SegResNet)	87%	0.78 (0.61)	0.56 (0.32)	0.73 (0.53)
MC dropout (*p* = 0.2)	66%	0.79 (0.63)	**0.73 (0.54)**	0.74 (0.54)
MC dropout (*p* = 0.5)	72%	0.81 (0.65)	**0.73 (0.54)**	**0.74 (0.55)**
Mixed Arch. (EDL)	**93%**	**0.82 (0.68)**	0.71 (0.50)	0.63 (0.40)

*p* refers to the dropout rate. Training details, including those for MC Dropout, and definitions of the evaluation metrics are provided in the Supplementary Information. All metrics were computed on the 68 segmentations in the test set.

Best values per metric are shown in bold.

First, we assessed the impact of architectural diversity by comparing the homogeneous and heterogeneous EDL ensembles. The homogeneous EDL using five SegResNets demonstrated 87% coverage and strong correlations with segmentation (*R*² = 0.61) and volumetric error (*R*² = 0.53). In comparison, the heterogeneous mixed architecture EDL ensemble demonstrated the benefits of architectural diversity. It achieved the highest coverage (93%), reflecting improved calibration, and exhibits stronger correlations with geometric error (*R*² = 0.50 vs. 0.32) and segmentation error (*R*² = 0.68 vs. 0.61). These findings indicate that combining diverse architectures enhances the model’s ability to capture spatial and volumetric deviations through its uncertainty estimates.

Next, we compared our proposed mixed architecture EDL ensembles to the MC Dropout baseline. While MC Dropout showed strong error correlations (*R*² = 0.65 for segmentation error, *R*² = 0.54 for ASSD), the mixed architecture EDL ensemble achieved comparable performance (*R*² = 0.68, *R*² = 0.50) while providing substantially better coverage (93% vs. 72%). This higher coverage indicates that EDL avoids the overconfidence of MC Dropout, producing more reliable credible intervals. In clinical practice, this improved calibration is critical for reducing false negatives, thereby supporting safer decision-making.

**Fig. 3:**
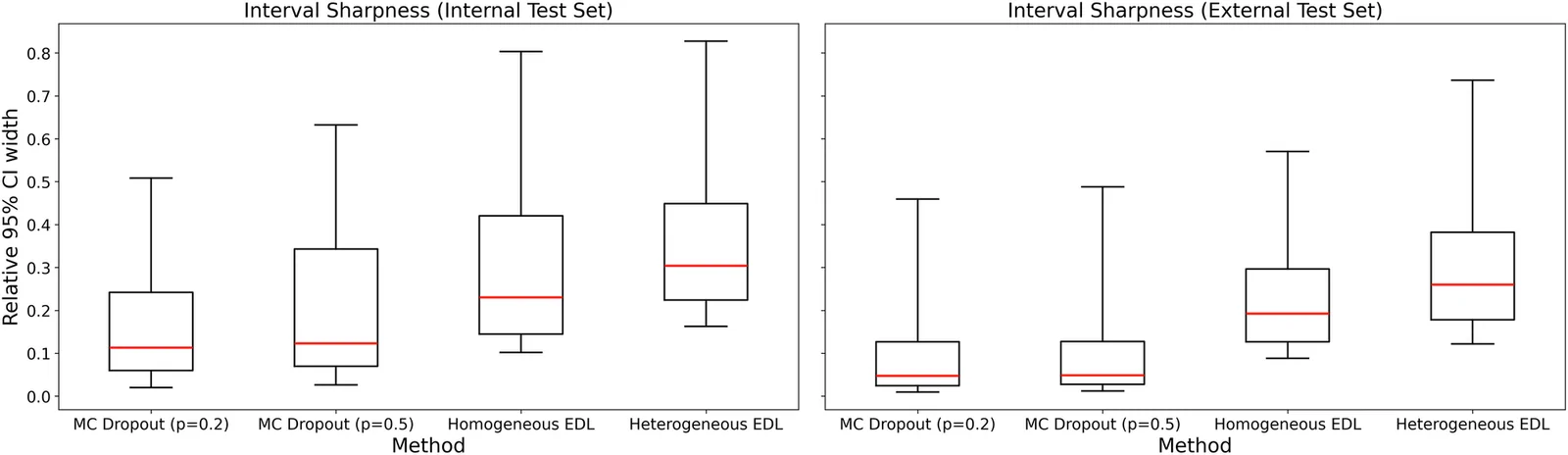
Interval sharpness comparison across methods on internal and external test sets. Boxplots show the distribution of volume-normalized 95% credible interval widths for MC Dropout (*p* = 0.2, *p* = 0.5), Homogeneous EDL, and Heterogeneous EDL. On the internal test set, MC Dropout yields the sharpest intervals with lower medians and tighter dispersion, while both EDL variants produce broader intervals. On the external test set, interval widths preserve the same ordering.

To evaluate the trade-off between calibration and sharpness, we additionally compared normalized 95% interval widths across methods (Fig. 3). MC Dropout produced the narrowest intervals but exhibited substantial under-coverage, whereas EDL ensembles generated moderately wider intervals while achieving markedly improved empirical coverage. This result indicates that the improved calibration of EDL is not solely due to excessive interval inflation but reflects more reliable uncertainty quantification.

Importantly, these findings generalize to the external cohort. Calibration decreased across institutions for all models, consistent with the expected impact of domain shift. Specifically, empirical coverage dropped to 60.6% for the homogeneous EDL ensemble and 72.8% for the heterogeneous EDL ensemble, compared to 17.6–20.7% for MC Dropout. Despite this reduction, both EDL configurations remained substantially less overconfident than MC Dropout under external distribution shift. Notably, the external dataset underwent skull-stripping during preprocessing, which differs from the internal test set and may partially contribute to the observed decrease in coverage across all methods.

To further assess whether interval width meaningfully reflects predictive reliability, we examined the relationship between predicted uncertainty and segmentation performance by analyzing median relative volumetric error (defined as the median absolute tumor volume error normalized by ground-truth volume) across bins of normalized 95% interval width (Figs. S6-S7). Across both datasets, all methods demonstrated a positive association between interval width and volumetric error, indicating that larger predicted uncertainty corresponds to higher segmentation error. The EDL ensembles exhibited a more consistently monotonic stratification across bins, whereas MC Dropout showed greater dispersion and partial overlap between uncertainty levels, particularly under external validation. These findings support that EDL provides more stable uncertainty–error coupling.

### Uncertainty-aware segmentation is qualitatively meaningful

**Fig. 4:**
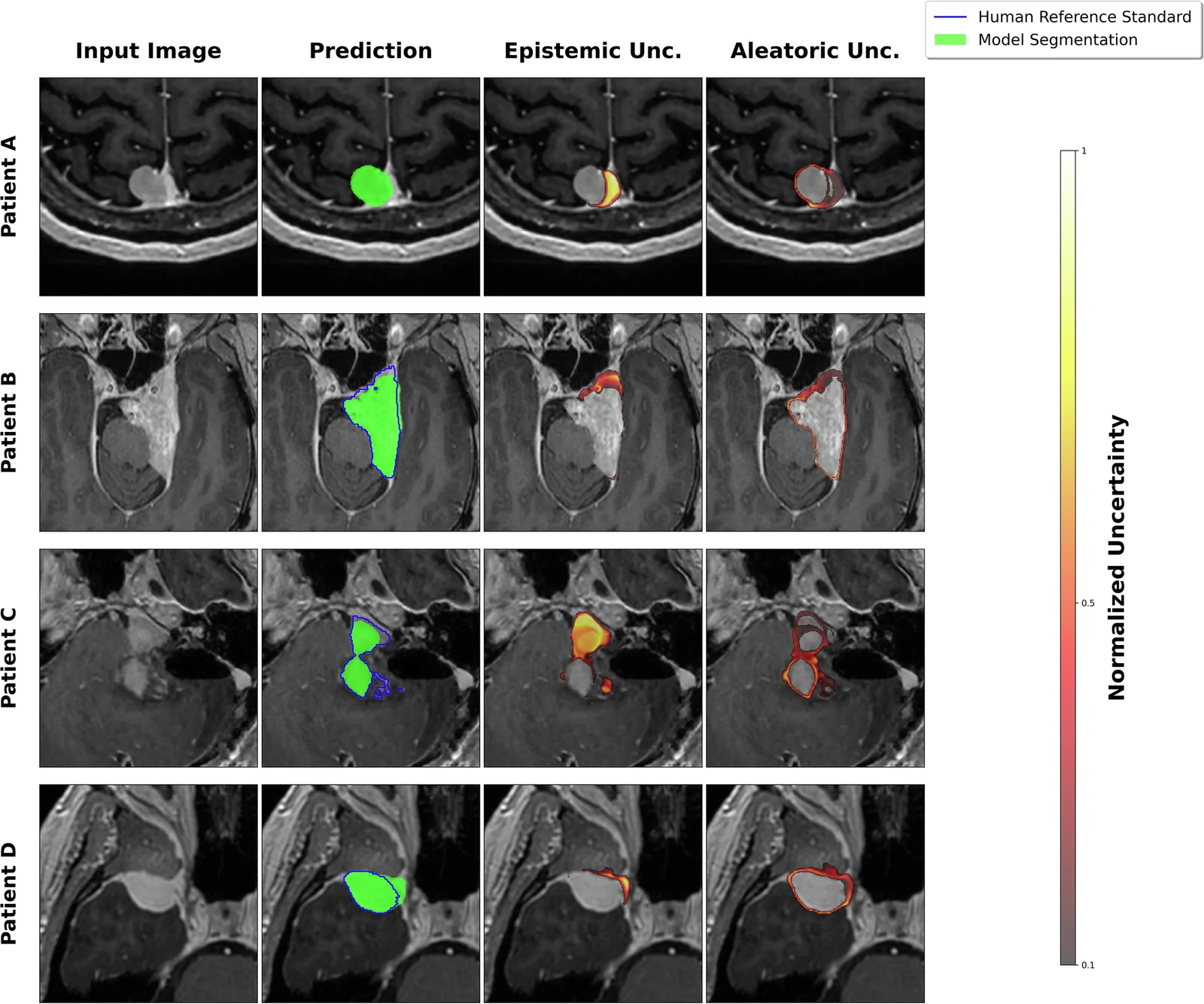
Qualitative visualization of the uncertainty-aware segmentation results for four challenging (difficulty score ≥7/10 as rated by a neuroradiologist) meningioma cases. Each panel shows a spatial decomposition of predictive uncertainty into epistemic and aleatoric components on a representative slice of the 3D input volume. Columns show, from left to right: the input image, the model prediction, the epistemic uncertainty map, and the aleatoric uncertainty map. The model segmentation is shown as a green overlay and the human reference standard as a blue contour, and the uncertainty maps are colour-coded according to the normalized uncertainty scale on the right (0.1–1). Aleatoric uncertainty systematically concentrates along tumor boundaries, aligning with regions of intrinsic voxel-level ambiguity, while epistemic uncertainty appears more spatially clustered, often localizing to anatomically ambiguous regions such as tumor-brain interfaces. Patient A’s tumor is challenging because of involvement of the adjacent superior sagittal sinus, which also enhances similarly to the meningioma. Patient B’s tumor is challenging because of involvement of the adjacent cavernous sinus, which enhances similarly to the meningioma. Patient C is a post-operative brain MRI with residual/recurrent meningioma, but some of the enhancing structures represent normal vasculature or post-operative scar tissue, making differentiation from residual tumor difficult. Patient D’s tumor extends along the dural reflection towards the sphenoparietal sinus, which is difficult to distinguish from the tumor boundary.

The novel mixed architecture EDL ensemble model produced realistic uncertainty-aware segmentation outputs (Fig. 4). As detailed in the “Methods”, the framework decomposes total uncertainty into two distinct components. Aleatoric-like uncertainty captures ambiguity intrinsic to the task itself, identifying voxels that are inherently difficult to classify regardless of the model’s training. Epistemic-like uncertainty reflects the model’s internal lack of knowledge, arising when it encounters anatomy that differs from the patterns seen in its training data. In challenging meningioma cases, we found that both the epistemic-like and aleatoric-like uncertainty estimates were high along the borders of the tumor, in particular at boundaries with other enhancing structures. The epistemic-like uncertainty maps reflect regions of uncertainty caused by the interface of enhancing tumor with other anatomically normal enhancing structures, such as the dural venous sinuses or post-operative changes. Aleatoric-like uncertainty, meanwhile, highlights the inherent uncertainty in the boundary of tumors in MRI data (e.g., due to partial voluming). Credible intervals tended to be wider for cases judged as difficult to segment by a neuroradiologist (Fig. S8), although there was no significant difference, likely due to insufficient data for very difficult cases.

**Fig. 5:**
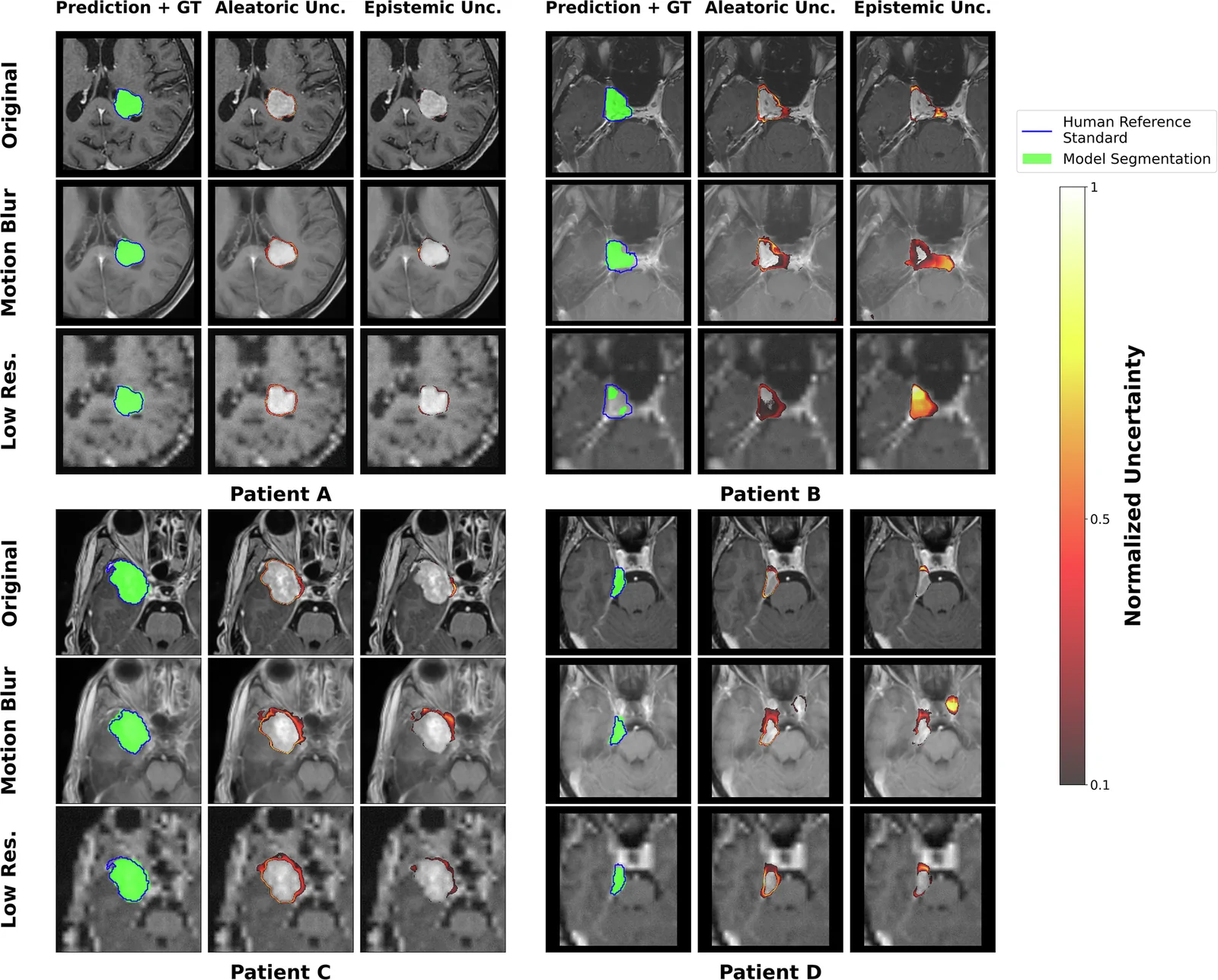
Qualitative visualization of segmentation robustness and uncertainty decomposition under image degradation for four MRIs with meningiomas. Each MRI is shown under three imaging conditions: original, motion blur, and low resolution, with columns depicting (i) prediction overlaid with ground truth, (ii) aleatoric uncertainty, and (iii) epistemic uncertainty. The human reference standard segmentation is shown in blue and the model prediction in green overlay. Uncertainty maps are colour-coded according to the normalized uncertainty scale on the right (0.1–1). For an easier case such as *Patient A*, both the segmentation and uncertainty maps remain stable across degraded inputs, indicating strong model confidence despite image degradations. For *Patients C* and *D*, degradation leads to visibly increased uncertainty without significantly affecting segmentation accuracy, consistent with the model expressing higher uncertainty while maintaining performance. In contrast, *Patient B*, a particularly challenging case of a cavernous sinus meningioma involving complex anatomy, shows both elevated uncertainty and a clear drop in segmentation accuracy under motion blur and low resolution. These results confirm the expected relationship: as real-world image artifacts increase task difficulty, the model becomes more uncertain, reflecting appropriate calibration of predictive confidence.

Next, to assess robustness under image acquisition variability, we simulated clinically plausible degradations such as motion blur and reduced resolution (Fig. 5). These degradations commonly arise in routine clinical workflows. Despite these perturbations, the model remained qualitatively robust, with uncertainty increasing appropriately under both conditions. Specifically, for well-defined, high-contrast tumors with sharp borders (Patient A), segmentation and uncertainty maps remained stable. In more ambiguous cases (Patients B–D), degradations amplified uncertainty in a clinically coherent manner. Thus, qualitatively, the uncertainty maps appear to offer a reliable signal of reduced confidence in the setting of image degradations, specifically in cases that require higher quality images.

### Utility of uncertainty in longitudinal evaluations of brain tumors

**Fig. 6:**
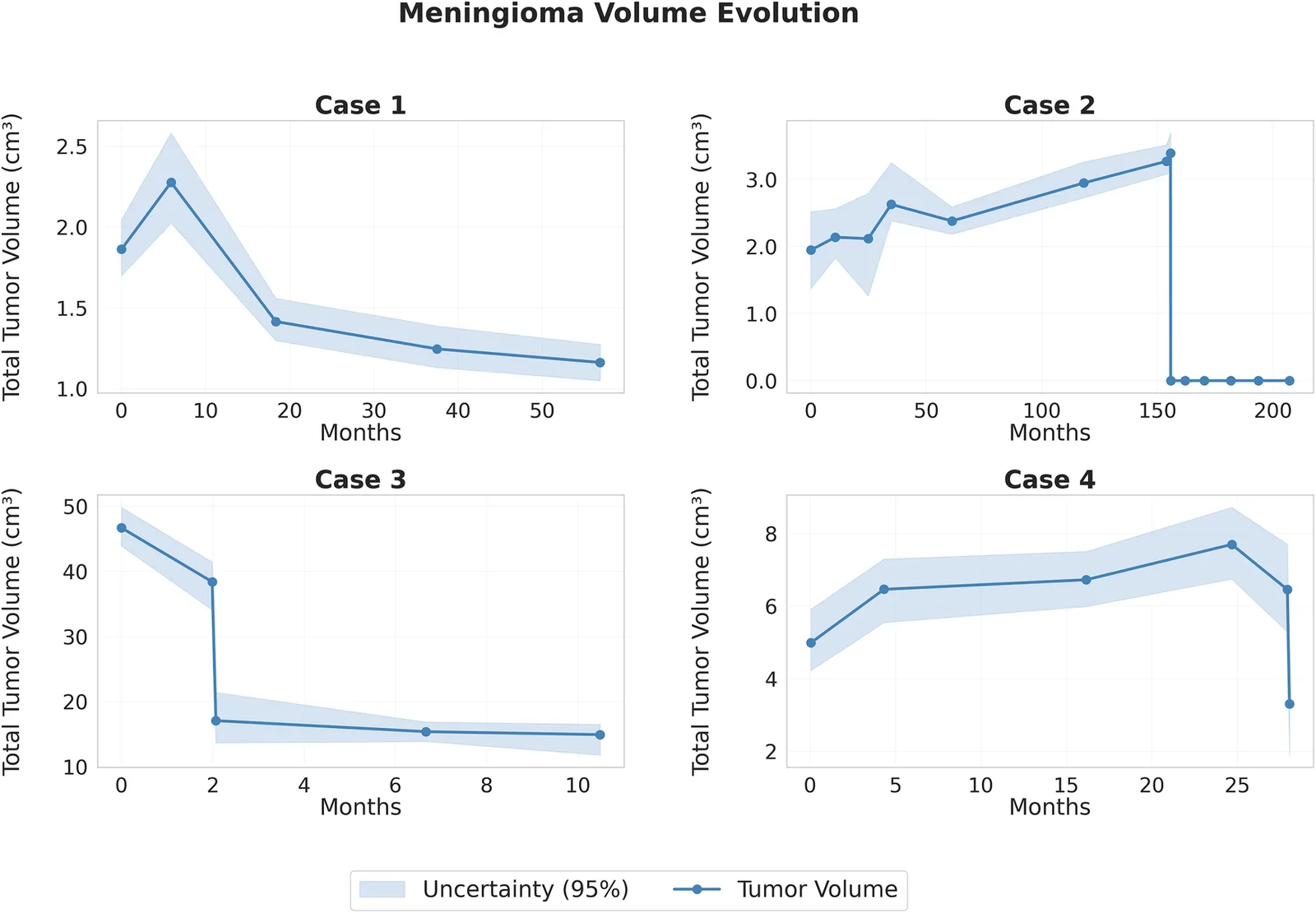
Longitudinal meningioma volume evolution with calibrated 95% credible intervals across four representative clinical scenarios. **Case** 1 demonstrates a post-radiation decrease after a brief period of fast growth. The post-radiation volume falls outside the pre-treatment credible interval, consistent with a true treatment response. **Case 2** shows growth of a tumor over 150 months followed by gross total resection with no residual tumor, where the post-operative volume is zero, reflecting a definitive structural change. **Case 3** illustrates partial resection followed by stability, with subsequent overlapping intervals consistent with stable disease. **Case 4** depicts subtotal resection after a period of slow growth over 25 months, where the volume decrease exceeds uncertainty bounds, indicating a clinically meaningful change. All these cases are consistent with neuroradiological assessment.

The above results demonstrate the utility of uncertainty estimates in guiding physicians to investigate areas of tumor segmentations that may be less reliable or where model confidence is low. To demonstrate the potential utility of uncertainty estimation in a longitudinal setting, voxel-wise uncertainty maps are translated into 95% credible intervals around volumetric measurements of the tumors (Fig. 6). These credible intervals endow longitudinal variation in volumes with interpretability, differentiating measurement noise from true changes in tumor volume.

Additional results and subgroup analyses are reported in the Supplementary Information.

## Discussion

In this work, we developed and validated an uncertainty-aware framework for meningioma volumetrics. Our approach, using evidential deep learning (EDL) ensembles, successfully produced principled credible intervals for tumor volume while maintaining state-of-the-art segmentation accuracy. By directly modeling the amount of evidence supporting the prediction at every voxel in the image, the EDL framework enables neural networks to express both what they predict and how confident they are. In the context of meningiomas, the most common primary brain tumor, our validation of this method demonstrates state-of-the-art segmentation accuracy with robust, meaningful uncertainty metrics. This capability has the potential to substantially increase trust in biomedical image segmentation, particularly in applications such as brain tumor volumetrics, where decisions are sensitive to boundary ambiguities and subtle longitudinal changes in the context of heterogeneous image quality.

The quality of uncertainty estimates is central to clinical applicability^18^. In our analysis, the heterogeneous EDL ensemble achieved the highest empirical coverage (92.8%), indicating that the reported credible intervals around the predicted tumor volumes reliably reflect the true variability of tumor volume estimates. This improved coverage was associated with wider credible intervals compared with MC dropout, reflecting a trade-off between calibration and sharpness. Moreover, error metrics of segmentations were correlated with the degree of uncertainty. In practice, uncertainty maps can help physicians focus their attention on challenging regions, improving efficiency in clinical review. Similarly, longitudinal credible intervals enable clinicians to distinguish true volumetric change from model ambiguity, supporting more reliable monitoring of tumor progression.

The voxel-level decomposition into epistemic and aleatoric uncertainty is visually interpretable and qualitatively aligns with regions where annotation variability would be expected, effectively translating theoretical uncertainty modeling into a form usable for radiological assessment. The epistemic-like component tended to be high in regions of uncertainty due to enhancing tumor abutting other normally enhancing structures, such as the dural venous sinuses. These are regions where it can be difficult for an expert neuroradiologist to differentiate between tumor and normal tissue. The aleatoric-like component tended to be high along the boundaries of the tumor, which is inherently uncertain. Motion blur and lower resolution qualitatively increased both components, most notably for ambiguous or unusual regions of tumors, where high-quality imaging is most essential. For clinical deployment, a unified uncertainty interval is ultimately more practical than separating epistemic and aleatoric components; thus, the present decomposition should be considered primarily illustrative. Future studies should evaluate its clinical role, as component-wise separation may be more relevant for model development and data selection.

A key finding of this study is that uncertainty quantification can be achieved without a trade-off in performance. Both homogeneous (Dice 0.95) and heterogeneous EDL (Dice 0.93) ensembles maintained high Dice scores comparable to or better than standard state-of-the-art segmentation networks, such as nnU-net v2 (Dice 0.77), confirming that uncertainty modeling need not come at the expense of segmentation accuracy. While the homogeneous ensemble achieved slightly higher median Dice, the heterogeneous ensemble provided superior calibration and stronger correlations with segmentation and volumetric error metrics. These results highlight the benefit of combining various architectures with distinct inductive biases to better capture complementary aspects of model uncertainty.

Compared to MC Dropout^19^, the evidential framework offers substantial computational advantages. MC Dropout requires 20 stochastic forward passes, resulting in significant runtime overhead, whereas the EDL ensembles produce uncertainty estimates in a single forward pass. This efficiency is particularly important for clinical deployment, where rapid inference and integration into existing workflows are essential.

While our current system was designed for deployment within the local workflow and trained exclusively on internal private data, the high segmentation performance on an independent external test set provides compelling evidence of cross-institutional generalization, without any fine-tuning. Nevertheless, future studies incorporating multi-center datasets and multi-rater annotations will be necessary to fully validate model uncertainty against human inter-observer variability.

Limitations of this study should be noted. While we compared the homogeneous and heterogeneous EDL frameworks as described, other combinations of ensemble models could achieve even higher performance. Moreover, it is unclear how to quantitatively compare uncertainty maps across methods and relate those to human uncertainty. Ultimately, future studies incorporating uncertainty quantification techniques like the one described here should measure these methods’ impact on radiological workflows and clinical decision making, which was beyond the scope of the current analyses.

In conclusion, we have presented an evidential deep learning framework that produces well-calibrated credible intervals for tumor volume. We found that leveraging architectural diversity within the ensemble is a powerful strategy for achieving high-quality uncertainty estimates. This work advances the broader goal of developing transparent, safe, and trustworthy AI for medicine, paving the way for uncertainty-aware quantitative monitoring of tumor dynamics in routine clinical care.

## Methods

### Study population

This retrospective study was approved by the University of California, San Francisco Institutional Review Board (IRB#18-24775) and the Duke University Institutional Review Board (IRB#Pro00110695), with a waiver of informed consent due to the minimal-risk, retrospective nature of the study. All procedures complied with the Declaration of Helsinki and the Health Insurance Portability and Accountability Act (HIPAA). Data were stored on secure, access-controlled servers and de-identified as necessary. The patient population has not been previously reported.

The study involved three distinct patient cohorts: a training cohort used for model development and validation, an independent test cohort for final evaluation, and an independent external cohort to assess model generalization.

The training cohort consisted of 1655 3D post-contrast T1-weighted brain MRIs (1324 for training and 331 for validation) from 788 unique patients from a curated private institutional dataset.

The ground truth segmentations for this cohort were generated through a semi-automated process. An initial nnUnet model^17^ was trained on a subset of the data to generate predictions on the full training set. During a subsequent curation step, segmentation outputs containing clearly erroneous regions were identified and manually corrected. The underlying MRIs were not excluded; rather, only clearly incorrect portions of the automated segmentations were removed prior to inclusion in the training set.

The independent test cohort consisted of 93 3D T1-weighted post-contrast brain MRIs from 55 unique patients (11 male, 44 female; age range: 35-85 years; mean age: 59 years). Patients included in the test cohort were not included in the training cohort. Exams with meningioma volumes smaller than 0.3 cm³ were excluded, as precise volumetric measurement and credible intervals at this scale are not clinically meaningful. After filtering, 68 MRIs from 43 unique patients (10 male, 33 female; age range: 35–82 years; mean age: 58 years) were retained for analysis.

For this cohort, the ground truth segmentation was reviewed and manually corrected on a voxel-wise basis by a neuroradiologist with 6 years of post-graduate clinical experience, directly guided and supervised by a neuroradiologist with 12 years of post-graduate clinical experience. Challenging cases were discussed in detail to create accurate reference standard segmentations.

The independent external institution test cohort consisted of 353 unique patients from Duke University [94 male, 259 female; age range: 19–96 years; mean age: 63.2 years]^20^, and is partially publicly available.

### Beta modeling of class probabilities

We adopt an Evidential Deep Learning (EDL) framework to model both predictive performance and voxel-wise uncertainty in a principled Bayesian setting. Unlike conventional softmax-based networks that output deterministic class probabilities, EDL models generate parameters of a predictive distribution, enabling uncertainty quantification that propagates to higher-level volumetric measures.

Let $x\in {R}^{H\times W\times D}$ denote the input 3D image volume and ${y}_{v}\in \mathrm{0,1}$ the binary voxel-level label indicating the presence or absence of tumor tissue. In our framework, the network, parameterized by θ, is trained to output parameters of a Beta distribution for each voxel v, as ${p}_{v}=p\left({y}_{v}=1,|,x,\theta \right)$. This is a special case of the Dirichlet distribution when K=2 classes.

Specifically, the neural network outputs two non-negative evidence values $\left({e}_{0},{e}_{1}\right)\in {R}_{+}^{2}$, which are passed through a ReLU activation to ensure positivity. The parameters of the Beta distribution are then defined as:

1 $${p}_{v}\sim {Beta}\left({\alpha }_{0},{\alpha }_{1}\right),{with}{\alpha }_{0}={e}_{0}+1,{\alpha }_{1}={e}_{1}+1$$

This formulation produces a predictive distribution over ${p}_{v}$ that reflects the model’s confidence in voxel-wise classification. The mean of this distribution represents the expected tumor probability given the input and the trained model:

2 $$E\left[{p}_{v},|,x,\theta \right]=\frac{{\alpha }_{1}}{{\alpha }_{0}+{\alpha }_{1}}$$

A binary segmentation mask is obtained by thresholding this probability at τ=0.5.

Furthermore, the model’s confidence in each voxel prediction is quantified by the variance of the Beta distribution:

3 $$V\left[{p}_{v},|,x,\theta \right]=\frac{{\alpha }_{0}{\alpha }_{1}}{{({\alpha }_{0}+{\alpha }_{1})}^{2}({\alpha }_{0}+{\alpha }_{1}+1)}$$

This captures within-model uncertainty, reflecting both noise in the input data and class overlap, given a fixed trained model ${\theta }^{\star }$.

### Loss function and optimization objective

To train the model to express reliable uncertainty and accurate segmentation, we use a compound loss function that balances prediction accuracy with uncertainty regularization:

4 $${L}_{\mathrm{EDL}}={L}_{\mathrm{Dice}}+\lambda \left(t\right)\cdot {L}_{\mathrm{KL}}$$

The first term, L_Dice_, focuses on segmentation accuracy. It is a variant of the standard Dice loss that rewards accurate spatial overlap between the prediction and the ground truth, while also penalizing the model for being uncertain about its correct predictions. The second term, *L*_KL_, is a regularization penalty that specifically discourages the model from making overconfident errors.

The mathematical formulation of these loss components and the annealing schedule *λ*(*t*) used to balance them during training are detailed in the Supplementary Information for technical completeness.

### Model ensembling and total uncertainty quantification

A single deep learning model, even one trained with EDL, can sometimes be overconfident. To obtain a more robust and reliable uncertainty estimate, we used an ensemble approach. Instead of relying on one model, we trained five independent models and aggregated their predictions. This strategy is analogous to seeking a consensus from multiple experts. It provides a practical and effective way to approximate the true Bayesian predictive distribution, which formally accounts for uncertainty over the model’s parameters. The mathematical basis for this approximation is detailed in the Supplementary Information.

Each model (m) yields a Beta distribution $\left({\alpha }_{0}^{\left(m\right)},{\alpha }_{1}^{\left(m\right)}\right)$ for each voxel v. The total predictive variance per voxel can then be decomposed into two components:

5 $$V\left[{p}_{v},|,x,D\right]={E}_{m}\left[V\left({p}_{v},|,{\theta }^{\left(m\right)},x\right)\right]+{V}_{m}\left[E\left({p}_{v},|,{\theta }^{\left(m\right)},x\right)\right]=\mathrm{Aleatoric}-\mathrm{like}\mathrm{uncertainty}+\mathrm{Epistemic}-\mathrm{like}\mathrm{uncertainty}$$

In this decomposition, the first term, often referred to as aleatoric-like uncertainty, captures the average intrinsic variance within each model’s prediction. This reflects the uncertainty that remains even with a single, perfectly trained model, such as from data noise or ambiguous voxel-level boundaries. It is typically considered irreducible with more data, as it is a property of the data-generating process itself.

The second term, referred to as epistemic-like uncertainty, measures the variance in the mean predictions across the ensemble. This represents the uncertainty arising from model parameter variability due to a finite training set. It manifests as disagreement among ensemble members and is considered reducible with the addition of more training data. To assess the impact of model diversity on the quality of uncertainty estimates, we evaluate two distinct configurations: a homogeneous ensemble composed of models with a single architecture, and a heterogeneous ensemble combining multiple, diverse architectures.

We use the terms “aleatoric-like” and “epistemic-like” to acknowledge that while this decomposition provides a valuable and intuitive framework for separating different sources of error, the strict philosophical dichotomy between aleatoric and epistemic uncertainty is a subject of ongoing debate in the literature, and they can be highly correlated in practice^21^. For instance, high aleatoric uncertainty at a difficult boundary might lead to greater model disagreement (epistemic uncertainty) as different models converge to slightly different solutions.

Nevertheless, this formulation provides a highly interpretable and clinically useful visual decomposition of uncertainty. It allows us to distinguish between regions of intrinsic ambiguity (e.g., tumor boundaries) and areas where the model is simply “unsure” due to a lack of training data or out-of-distribution input (e.g., dural reflections), which is especially important for downstream tasks such as volumetric quantification.

### Bayesian estimation of tumor volume with credible intervals

To derive uncertainty-aware tumor volumes, we employ Bayesian Model Averaging (BMA) over the ensemble of voxel-wise Beta distributions predicted by our models. Given the ensemble, we define a mixture model over class probabilities and compute credible intervals through Monte Carlo sampling. For each voxel v, we first compute the expected tumor probability for each model:

6 $${p}_{v}^{\left(m\right)}=\frac{{\alpha }_{1}^{\left(m\right)}}{{\alpha }_{0}^{\left(m\right)}+{\alpha }_{1}^{\left(m\right)}}$$

The ensemble mean prediction is then obtained by averaging over the M models:

7 $$\widehat{{{\rm{p}}}_{{\rm{v}}}}=\frac{1}{{\rm{M}}}\mathop{\sum }\limits_{{\rm{m}}=1}^{{\rm{M}}}{{\rm{p}}}_{{\rm{v}}}^{\left({\rm{m}}\right)}$$

However, the ensemble mean alone is insufficient to construct calibrated volumetric credible intervals, as it collapses the full posterior distribution to a point estimate and does not capture its dispersion. We therefore propagate posterior variability by sampling from the mixture distribution, enabling quantile-based interval estimation.

To capture predictive uncertainty, we sample voxel-wise class probabilities from the mixture of Beta distributions defined by the ensemble. Concretely, for each voxel v, we draw N samples:

8 $${p}_{v}^{\left(n\right)}\sim Beta\left({\alpha }_{0}^{\left({m}_{n}\right)},{\alpha }_{1}^{\left({m}_{n}\right)}\right)$$

where ${m}_{n}$ is randomly selected from 1,…,M. We selected N=1000 as it provides a stable estimate of the posterior distribution without excessive computational overhead. From the N samples, we estimate the γ-credible interval by computing the quantiles ${q}_{\left(1-\gamma \right)/2}$ and ${q}_{\left(1+\gamma \right)/2}$.

Based on these quantiles, we define three segmentation masks:

- Baseline segmentation ${S}_{\mathrm{base}}$: voxel v is included if ${\hat{p}}_{v} > 0.5$.
- High confidence segmentation ${S\left(\gamma \right)}_{\mathrm{high}\mathrm{conf}}$: voxel v is included if ${q}_{\left(1-\gamma \right)/2} > 0.5$, indicating high confidence in tumor presence.
- Inclusive segmentation ${S\left(\gamma \right)}_{\mathrm{inc}}$: voxel v is included if ${q}_{\left(1+\gamma \right)/2}\ge 0.5$, indicating even marginal suspicion of tumor.

These three masks allow us to define a credible interval for the total tumor volume:

9 $${V}_{highconf}\le {V}_{base}\le {V}_{inc}$$

where each volume $V={\sum }_{v}{S}_{v}\Delta x\Delta y\Delta z$ is computed by summing over the binary segmentation mask ${S}_{v}$ and multiplying by the voxel spacing for each dimension, Δx, Δy and Δz.

### Models training

To construct a diverse ensemble, we implemented a principled strategy designed to induce variation across multiple orthogonal axes to improve the robustness and calibration of uncertainty estimates. Our approach systematically introduces diversity^22^ at the data, model, and objective levels:

- Data-level diversity**:** We used a 5-fold cross-validation structure combined with bootstrap resampling of the training data for each fold. This ensures that each model is trained on a unique, though overlapping, subset of the patient data, exposing it to different sampling distributions.
- Model-level diversity**:** Each model was trained from a distinct random initialization. Furthermore, each network was subjected to its own unique sequence of data augmentations during training, including random flips, rotations, and intensity shifts, forcing them to learn along different optimization trajectories.
- Objective-level diversity**:** We varied the hyperparameters of the loss function for each model, specifically the weight and annealing schedule of the KL-divergence penalty. This encourages individual models to learn different trade-offs between segmentation accuracy and uncertainty penalization. We also modulated the softness of the ground-truth boundary on a per-model basis (by dilating the tumor mask by 0–4 voxels when computing the regularization loss), prompting different models to learn varying degrees of caution at ambiguous tumor margins.

Our study compares two main ensemble configurations built from three different deep learning architectures 3D SegResNet^23^, SWIN UNETR^24^, and DiNTS^25^:

- A homogeneous ensemble consisting of five 3D SegResNet models.
- A heterogeneous ensemble consisting of two 3D SegResNets, two SWIN UNETRs, and one DiNTS model.

The specific preprocessing, implementation, and training hyperparameters for each architecture are detailed in the Supplementary Information.

### Evaluation and statistical analysis

All evaluations were performed on the independent, anonymized test set. The primary outcome for assessing uncertainty was the *empirical coverage* of the 95% volumetric credible intervals. Secondary outcomes included model robustness, segmentation accuracy (Dice Similarity Coefficient)^26^, and the clinical relevance of the uncertainty estimates. To validate this clinical relevance, we assessed the model’s ability to predict its own failure modes, based on the principle that an ideal uncertainty estimate should be high when the model’s prediction is inaccurate. Accordingly, we investigated the relationship between the model’s predicted volumetric uncertainty and three distinct error metrics: Segmentation Error, Geometric Error (ASSD), and Relative Volumetric Error. Based on a preliminary analysis suggesting a power–law relationship, this was assessed using *Pearson correlation* on log-transformed variables, except for the Relative Volumetric Error, which was analyzed on a linear scale, with a *P* value of less than 0.05 considered statistically significant. The detailed mathematical definitions for the error metrics are provided in the Supplementary Information.

## Supplementary information

Supplementary information

## Data Availability

The datasets generated and/or analyzed during the current study are not publicly available due to institutional policies at UCSF and HIPAA privacy regulations protecting patient data but are available from the corresponding author on reasonable request. A subset of the external validation cohort is publicly available through the Brain Tumor Segmentation (BraTS) challenge dataset.

## Code availability

The custom code used for preprocessing, model training, inference, and uncertainty analysis in this study is publicly available at https://github.com/yassinegue/Meningioma-Seg. The implementation is written in Python and uses PyTorch (v2.6.0) and MONAI (v1.4.0) for development of the SegResNet-based segmentation models. Additional libraries for medical image processing and analysis include NumPy, SciPy, nibabel, and scikit-image. The repository provides scripts for MRI preprocessing, model training, ensemble inference, and uncertainty estimation. Detailed instructions, software dependencies, and training parameters used to generate the results reported in this study are documented in the repository.
