# Supplementary material for: Segmenting with confidence through uncertainty quantification for brain tumor imaging
Source: NPJ Digit Med. 2026 Jun 19;9:764. doi: 10.1038/s41746-026-02902-0 (PMC13639024; doi:10.1038/s41746-026-02902-0)
Supplement: Supplementary file 1 [file 41746_2026_2902_MOESM1_ESM.docx]

Supplementary Information

Loss function definition

The first term, $L_{\mathrm{Dice}}$, is a soft Bayesian Dice loss. It operates directly on the expected prediction $\hat{p}_{v}$ and penalizes high predictive variance $V\left[ p_{v} \right]$:

(1)

$$\mathcal{L}_{\text{Dice}}=1-\frac{2\sum_{v} y_{v}\hat{p_{v}}}{\sum_{v} y_{v}^{2}+\hat{p_{v}^{2}}+V\left[ p_{v} \right]}$$

This formulation encourages the model to produce high evidence (i.e., low variance) for correct predictions.

The second term, $L_{\mathrm{KL}}$, is a regularization loss that penalizes overconfident predictions on misclassified voxels. It computes the Kullback–Leibler divergence between the predicted Beta distribution and a uniform prior:

(2)

$$L_{\mathrm{KL}}=D_{\mathrm{KL}}\left[ \text{Beta}\left( \tilde{\alpha} \right)\parallel\text{Beta}\left( 1,1 \right) \right]=\log\left( \Gamma\left( \tilde{S} \right)/\Gamma\left( 2 \right) \right)-\sum_{c=0}^{1} \log\Gamma\left( \tilde{\alpha_{c}} \right)+\sum_{c=0}^{1} \left( \tilde{\alpha_{c}}-1 \right)\left[ \psi\left( \tilde{\alpha_{c}} \right)-\psi\left( \tilde{S} \right) \right]$$

where $\tilde{\alpha}=y_{one-hot}+\left( 1-y_{one-hot} \right)\odot\alpha$ and $\tilde{S}=\tilde{\alpha}_{0}+\tilde{\alpha}_{1}$. Here, $y_{one-hot}\in\left( 1,0 \right),\left( 0,1 \right)$ is the one-hot encoded ground truth label, and $\odot$ denotes element-wise multiplication. The use of $\tilde{\alpha}$ instead of $\alpha$ masks the contribution of the true class to focus the KL penalty on the incorrect one.

To balance the two terms during training, we apply an annealing schedule to $\lambda\left( t \right)$:

(3)

$$\lambda\left( t \right)=\frac{1}{n_{1}}\cdot min\left( 1,\left( \frac{t}{n_{2}} \right)^{2} \right)$$

This encourages the model to focus initially on learning discriminative features before penalizing overconfidence.

Bayesian Ensemble Approximation

In a full Bayesian framework, predictive uncertainty is derived from the posterior distribution $p\left( y | x,D \right)=\int p\left( y | x,\psi\right)p\left( \psi| D \right)d\psi,$ where $p\left( \psi\mid D \right)$ is the posterior over model parameters. As this integral is intractable, standard Evidential Deep Learning (EDL) methods approximate it by assuming a single point estimate for the optimal parameters, $\psi^{\star}$, which is formally equivalent to setting the model posterior to a Dirac delta function, $p\left( \psi\mid D \right)\approx\delta\left( \psi-\psi^{\star} \right).$This simplification, however, completely neglects the epistemic uncertainty associated with the model parameters themselves. Our work addresses this limitation by employing a deep ensemble of $M$ independently trained models $\{\psi^{\left( m \right)}{\}}_{m=1}^{M}$to serve as a Monte Carlo approximation of the intractable integral, thereby re-integrating this critical source of uncertainty:

(3)

$p\left( y \mid x,D \right)\approx\frac{1}{M}\sum_{m=1}^{M} p\left( y \mid x,\psi^{\left( m \right)} \right)$. Consequently, our combined approach provides a more comprehensive estimate of total epistemic uncertainty than standard single-model EDL, capturing both the uncertainty expressed by individual evidential models and the parameter uncertainty expressed through their disagreement.

Training details

The specific training configurations for each architecture were tailored to their computational requirements while promoting model diversity. SegResNet models were trained on volumes preprocessed to the dataset’s median voxel spacing using a random crop size of 296×296×128 voxels. SWIN UNETR and DiNTS models were trained on volumes resampled to a 1mm^3^ isotropic voxel spacing, with crop sizes of 160×160×160 and 128×128×128, respectively. These region of interest sizes were selected as the maximum possible for each architecture to avoid out-of-memory errors. At inference, full-resolution segmentations for all models were reconstructed via a sliding window strategy with Gaussian-weighted blending.

All models were implemented in the MONAI framework and shared a common training protocol. To manage memory demands, we used a per-GPU batch size of 1 with gradient accumulation over 4 steps, Group Normalization, and mixed-precision (FP16) training. Each model was trained for 1000 epochs using the AdamW ^27^ optimizer with a base learning rate of 2×10^−5^ and weight decay of 5×10^−4^, governed by a linear warm-up and cosine annealing schedule. The MC Dropout models, trained with dropout rates of 0.2 and 0.5, used the same training setup as the EDL variants, except that the EDL-specific loss functions were replaced by a standard Dice + Focal loss and the ReLU activation was substituted with a Softmax applied to the model’s logits.

Training was distributed across two NVIDIA Tesla V100 GPUs per model. The average epoch duration was approximately 12 minutes for SegResNet and SWIN UNETR, and 6 minutes for the computationally lighter DiNTS model. For inference, we retained the checkpoint corresponding to the highest validation Dice score for each model.

Quantitative metrics

The correlation analysis quantified the relationship between the model's predicted volumetric uncertainty and three distinct prediction error metrics. These metrics are defined as follows:

1. Predicted Volumetric Uncertainty: The relative width of the volumetric credible interval.

(4)

$$\frac{V_{\text{inc}}-V_{\text{high conf}}}{V_{\text{base}}}$$

2. Segmentation Error: The complement of the Dice Similarity Coefficient, measuring volumetric overlap error. Let P and G be the predicted and ground-truth voxel sets, respectively.

$$1-\frac{\left| P \right|+\left| G \right|}{2\left| P\cap G \right|}$$

(5)

3. Geometric Error (ASSD): The Average Symmetric Surface Distance between the predicted (P) and ground-truth (G) segmentations. Let S(⋅) be the set of surface voxels.

$\mathrm{ASSD}\left( P,G \right)$

(6)

$$=\frac{1}{2}\left( \frac{1}{\left| S\left( P \right) \right|}\sum_{p\in S\left( P \right)} \min_{g\in S\left( G \right)} \left( \left| \left| p-g \right| \right|_{2} \right)+\frac{1}{\left| S\left( G \right) \right|}\sum_{g\in S\left( G \right)} \min_{p\in S\left( P \right)} \left( \left| \left| g-p \right| \right|_{2} \right) \right)$$

4. Relative Volumetric Error: The absolute difference in predicted volume ($V_{P}$) and ground-truth volume ($V_{\mathrm{GT}}$), normalized by the ground-truth volume.

(7)

$$\frac{\left| V_{P}-V_{\mathrm{GT}} \right|}{V_{\mathrm{GT}}}$$

Quantitative evaluation of the homogeneous EDL ensemble

Figure S1. Quantitative evaluation of the homogeneous EDL ensemble on the test set (N=68). (A) Distribution of Dice scores, stratified by ground truth tumor volume. The model maintains high performance across a range of tumor sizes. The few zero-Dice points correspond to challenging or out-of-distribution cases: the green dot reflects the most difficult example (rated 9/10), while the others are small 4 cm³ tumors with resolutions differing from the training data. (B) Predicted versus ground truth volume, colored by radiologist-assessed difficulty. Error bars indicate the 95% credible interval derived through the EDL framework

Figure S2. Quantitative evaluation of the MC Dropout ensemble (p=0.2) on the test set (N=68). (A) Distribution of Dice scores, stratified by ground truth tumor volume. The model maintains high performance across a range of tumor sizes. The few zero-Dice points correspond to challenging or out-of-distribution cases: the green dot reflects the most difficult example (rated 9/10), while the others are small 4 cm³ tumors with resolutions differing from the training data. (B) Predicted versus ground truth volume, colored by radiologist-assessed difficulty. Error bars indicate the 95% credible interval derived through MC Dropout. While Dice performance is comparable to the EDL ensembles, the predictive intervals are noticeably sharper

Figure S3. Quantitative evaluation of the MC Dropout ensemble (p=0.5) on the test set (N=68). (A) Distribution of Dice scores, stratified by ground truth tumor volume. The model maintains high performance across a range of tumor sizes. The few zero-Dice points correspond to challenging or out-of-distribution cases: the green dot reflects the most difficult example (rated 9/10), while the others are small 4 cm³ tumors with resolutions differing from the training data. (B) Predicted versus ground truth volume, colored by radiologist-assessed difficulty. Error bars indicate the 95% credible interval derived through the EDL framework. Again, Dice scores are similar, but the intervals are substantially narrower

Dice score per tumor volume

A consistent performance trend is observed across both the internal UCSF (Figure S1) and external Duke (Figure S2) test sets. The model's segmentation accuracy, as measured by the Dice score, shows a clear positive correlation with tumor volume. For smaller tumors, particularly those in the 0.3-1 cm³ range, the model is more sensitive to error, exhibiting lower median Dice scores, greater variance, and a higher number of outlier cases with poor overlap. Conversely, as tumor volume increases, the model's performance becomes significantly more robust and reliable. For larger tumors (>5 cm³), the segmentation is highly accurate.

Figure S4: Segmentation Performance by Tumor Volume on the UCSF Dataset. Boxplots of Dice scores achieved by the model, stratified by ground truth tumor volume groups (cm³). The number of cases (n) in each group is indicated on the x-axis.

Figure S5: Segmentation Performance by Tumor Volume on the Duke Dataset.

Calibration and error correlation metrics on the external cohort

Table S1. Empirical coverage and Pearson correlations between predicted 95% credible interval width and segmentation metrics (segmentation error, average symmetric surface distance, and volumetric error) across four different framework, for the external cohort.

| Method | Coverage | Seg. Error ($R^{2}$) | ASSD ($R^{2}$) | Vol. Error ($R^{2}$) |
| --- | --- | --- | --- | --- |
| Homogeneous EDL (SegResNet) | 61 % | 0.84 (0.71) | 0.66 (0.44) | 0.61 (0.38) |
| MC Dropout (p=0.2) | 18 % | 0.77 (0.59) | 0.59 (0.35) | 0.53 (0.28) |
| MC Dropout (p=0.5) | 21 % | 0.42 (0.18) | 0.36 (0.13) | 0.45 (0.20) |
| Mixed Arch. (EDL) | 73 % | 0.80 (0.65) | 0.66 (0.43) | 0.30 (0.10) |

Uncertainty–Error Stratification Analysis

Figure S6. Relationship between interval width and volumetric error on the internal test set. Median relative volumetric error (IQR bars) is plotted against 95% credible interval width for three tumor-volume bins (small, medium, large) across methods. All methods show increasing error with increasing interval width, with EDL variants exhibiting clearer bin separation and more stable uncertainty–error stratification.

Figure S7. Relationship between interval width and volumetric error on the external test set. The same analysis performed on the external cohort. The monotonic increase in error with interval width is preserved, though variability increases across methods. EDL approaches maintain stronger stratification between uncertainty bins compared to MC Dropout, indicating more consistent alignment between predicted interval width and realized segmentation error under distributional shift.

Correlation with segmentation difficulty

To explore whether segmentation performance was associated with segmentation difficulty, a radiologist qualitatively rated each case on a scale from 1 to 10. Few cases received scores above 4, therefore, we grouped ratings 1–3 as “easy” and ratings 6 and above as “hard”, leading to a split of 53 easy cases and 8 hard cases. Qualitatively, a slight trend can be observed, with harder cases exhibiting marginally higher normalized credible interval widths (Figure S8, left) and lower Dice scores (Figure S8, right). However, Mann–Whitney U tests did not reveal significant differences (width U = 337.0, p = 0.65; Dice U = 477.0, p = 0.14). These non-significant results may reflect noise arising from the limited number of hard cases and the subjective bias of a single annotator. Future work with larger, independently rated datasets will be required to more robustly assess correlations between uncertainty and radiological difficulty.

Figure S8. Relationship between uncertainty width and radiologist-assessed difficulty for the heterogeneous EDL ensemble. Left: normalized credible interval width versus difficulty ratings. Right: Dice score versus difficulty ratings. Statistical interpretation remains limited by the class imbalance and small number of cases in the “Hard” difficulty group.
